# Supplementary material for: Tsc1 Loss in VIP-Lineage Cortical Interneurons Results in More VIP+ Interneurons and Enhanced Excitability
Source: Cells. 2023 Dec 26;13(1):52. doi: 10.3390/cells13010052 (PMC10777938; doi:10.3390/cells13010052)
Supplement: Supplementary file 1 [file cells-13-00052-s001.zip › Hu et al., Supp. files.pdf]

Supplementary Materials, Methods, and Data

Antibodies

Primary antibodies included mouse Calretinin (1:250, Catalog #6B3, Swant, Burgdorf, Switzerland) and goat SP8 (1:2000, Catalog #SC-104661, Santa Cruz Biotechnologies, Santa Cruz, USA).

In situ hybridization probes

Cb1 RNA probe was attained from the lab of Ivan Soltesz [1], and methods performed as described in main text.

TUNEL assay

Fluorescent TUNEL was performed according to manufacturer protocol (ApopTag Fluorescein In Situ Hybridization Apoptosis Detection kit, Catalog #S7110, MilliporeSigma, St. Louis, USA).

Statistics

Data were assessed in the same manner described in the main text. However, since several quantified data in supplemental files only included two groups, we used a Two-tailed T-test for these comparisons and a One-Way ANOVA for three or more comparisons, to assess significance for normally distributed data.

Figures and Legends

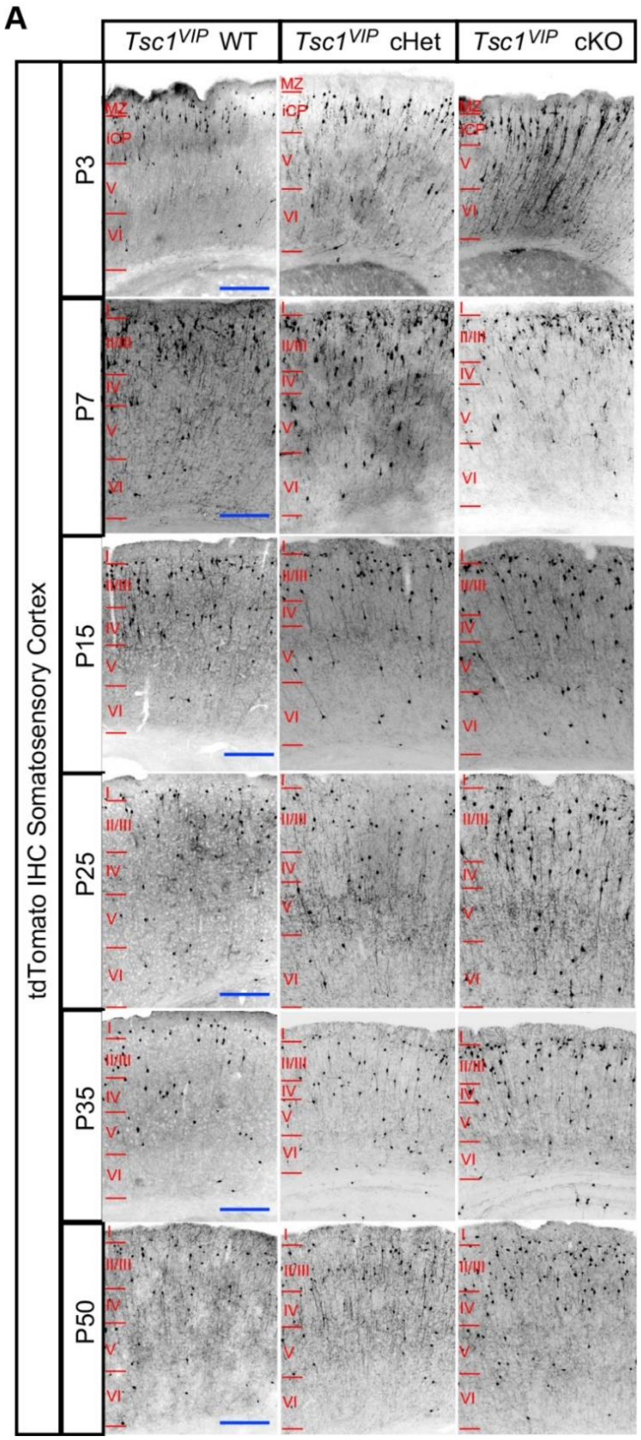

SUPPLEMENTAL FIGURE 1: TdTomato representative images in Tsc1 mutants over time. (A) tdTomato+ immunohistochemistry on P3, P7, P15, P25, P35, P50 wildtype (left), heterozygote (middle), and mutant (right) somatosensory cortices (n=8, 4, 8, 8, 4, and 4 for each genotype at P3, P7, P15, P25, P35, and P50 respectively). Cortical laminae are denoted by text and lines on the left side of each panel. Scale bars: 50μm.

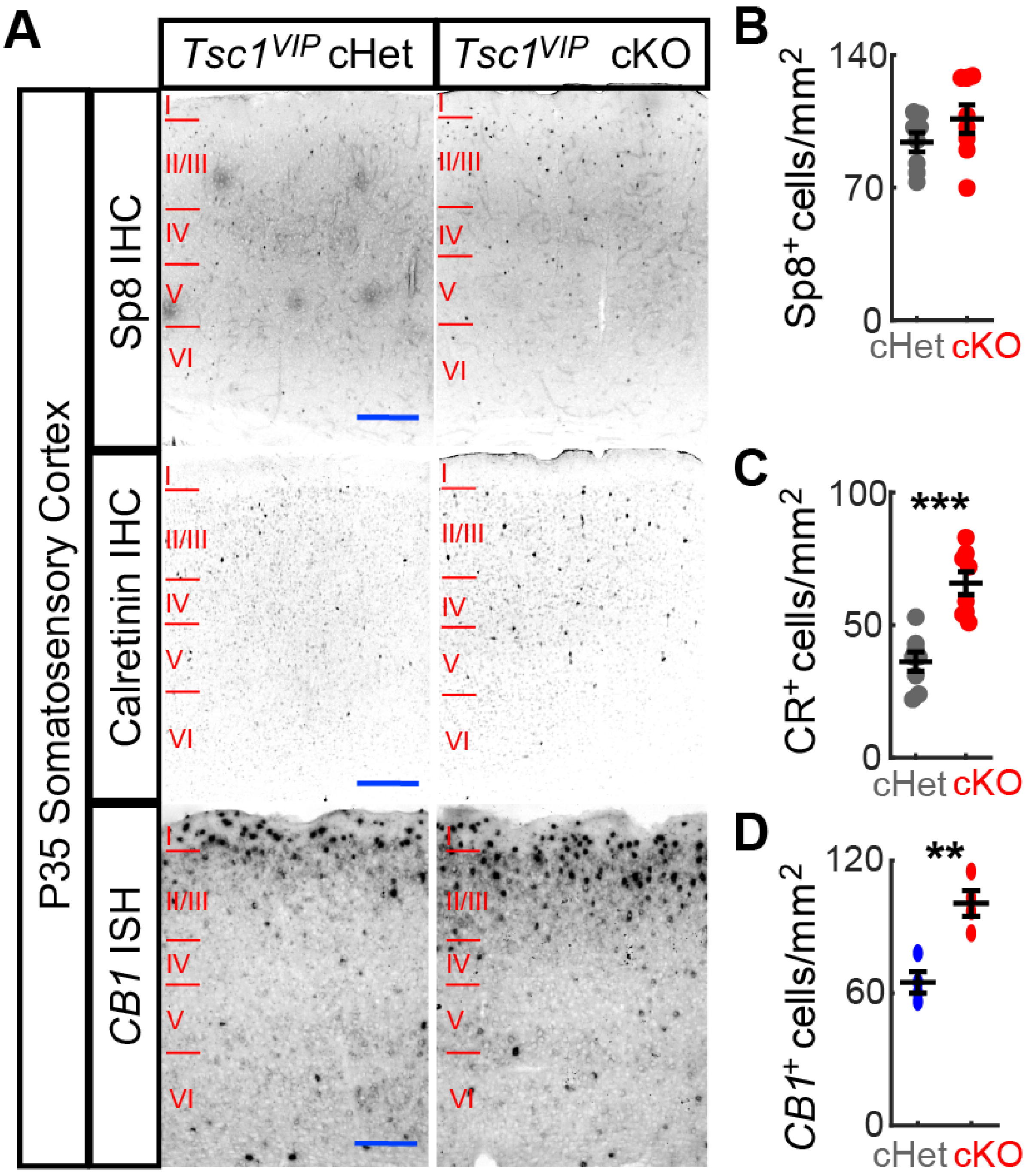

SUPPLEMENTAL FIGURE 2: (A) SP8 immunohistochemistry (top panels) (n=8 for each control and mutant), Calretinin immunohistochemistry (middle panels) (n=6-7 for each control and mutant), and CB1 in situ hybridization (bottom panels) (n-4 for each control and mutant) on P35 control (left column) and mutant (right column) somatosensory cortices. (B-D) SP8+ (B), Calretinin+ (C), and *CB1*+ (D) cell density quantifications for all layers in heterozygote (grey) and mutant (red) P35 somatosensory cortices. Scale bars: 50 μm. **p<0.01; ***p < 0.001.

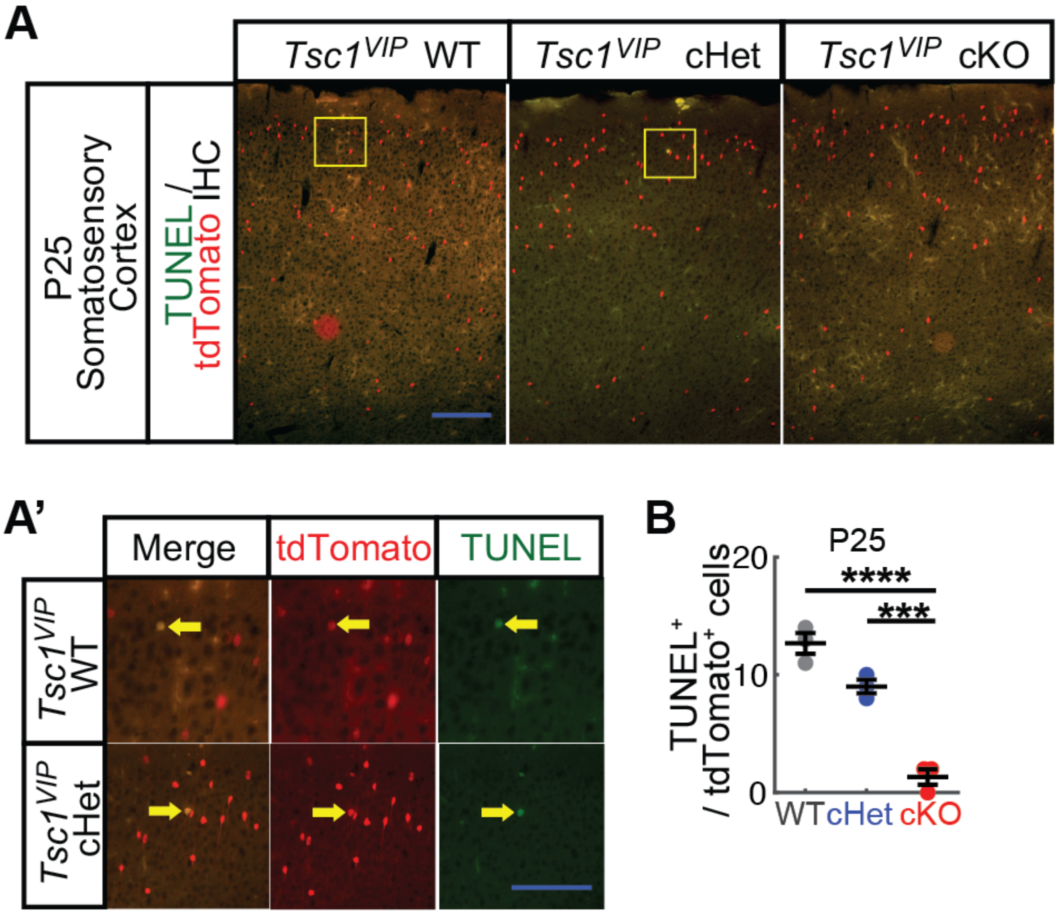

SUPPLEMENTAL FIGURE 3: (A) TUNEL+/tdTomato+ double labeling on P25 wildtype (left), heterozygote (middle), and mutant (right) somatosensory cortices. Scale bar: 50 µm. (A') Magnified views from yellow boxes in (A). Individual tdTomato (middle panel) and TUNEL (right panel) images are shown. Arrow points to VIP+ (tdTomato+) cell that are dying (TUNEL+). Scale bar: 25 µm. (B) Double TUNEL+/tdTomato+ cell counts for all layers from rostro caudal series of coronal P25 WT (grey), cHet (blue) and cKO (red) hemisections; n = 4 mice per genotype with over 3,000 tdTomato+ cells assessed per brain. Data are represented as mean +/- SEM. ***$p < 0.001$; ****$p < 0.0001$.

References

1. Varga, C.; Lee, S.Y.; Soltesz, I. Target-Selective GABAergic Control of Entorhinal Cortex Output. *Nat. Neurosci*. 2010, 13, 822–824, doi:10.1038/nn.2570.
